# Genome-wide gene expression analysis of a murine model of prostate cancer progression: Deciphering the roles of *IL-6* and *p38 MAPK* as potential therapeutic targets

**DOI:** 10.1371/journal.pone.0237442

**Published:** 2020-08-13

**Authors:** Reem Daouk, Hisham F. Bahmad, Eman Saleh, Alissar Monzer, Farah Ballout, Humam Kadara, Wassim Abou-Kheir

**Affiliations:** 1 Department of Anatomy, Cell Biology and Physiological Sciences, Faculty of Medicine, American University of Beirut, Beirut, Lebanon; 2 Arkadi M. Rywlin M.D. Department of Pathology and Laboratory Medicine, Mount Sinai Medical Center, Miami Beach, FL, United States of America; 3 Herbert Wertheim College of Medicine, Florida International University, Miami, FL, United States of America; 4 Department of Translational Molecular Pathology, The University of Texas MD Anderson Cancer Center, Houston, TX, United States of America; Medizinische Universitat Innsbruck, AUSTRIA

## Abstract

**Background:**

Prostate cancer (PCa) is the most commonly diagnosed cancer and the second leading cause of cancer-related deaths among adult males globally. The poor prognosis of PCa is largely due to late diagnosis of the disease when it has already progressed to an advanced stage marked by androgen-independence, thus necessitating new strategies for early detection and treatment. We construe that these direly needed advances are limited by our poor understanding of early events in the progression of PCa and that would thus represent ideal targets for early intervention. To begin to fill this void, we interrogated molecular “oncophenotypes” that embody the transition of PCa from an androgen-dependent (AD) to–independent (AI) state.

**Methods:**

To accomplish this aim, we used our previously established AD and AI murine PCa cell lines, PLum-AD and PLum-AI, respectively, which recapitulate primary and progressive PCa morphologically and molecularly. We statistically surveyed global gene expressions in these cell lines by microarray analysis. Differential profiles were functionally interrogated by pathways, gene set enrichment and topological gene network analyses.

**Results:**

Gene expression analysis of PLum-AD and PLum-AI transcriptomes (n = 3 each), revealed 723 differentially expressed genes (392 upregulated and 331 downregulated) in PLum-AI compared to PLum-AD cells. Gene set analysis demonstrated enrichment of biological functions and pathways in PLum-AI cells that are central to tumor aggressiveness including cell migration and invasion facilitated by epithelial-to-mesenchymal transition (EMT). Further analysis demonstrated that the p38 mitogen-activated protein kinase (MAPK) was predicted to be significantly activated in the PLum-AI cells, whereas gene sets previously associated with favorable response to the p38 inhibitor SB203580 were attenuated (i.e., inversely enriched) in the PLum-AI cells, suggesting that these aggressive cells may be therapeutically vulnerable to p38 inhibition. Gene set and gene-network analysis also alluded to activation of other signaling networks particularly those associated with enhanced EMT, inflammation and immune function/response including, but not limited to *Tnf*, *IL-6*, *Mmp 2*, *Ctgf*, and *Ptges*. Accordingly, we chose SB203580 and IL-6 to validate their effect on PLum-AD and PLum-AI. Some of the common genes identified in the gene-network analysis were validated at the molecular and functional level. Additionally, the vulnerability to SB203580 and the effect of IL-6 were also validated on the stem/progenitor cell population using the sphere formation assay.

**Conclusions:**

In summary, our study highlights pathways associated with an augmented malignant phenotype in AI cells and presents new high-potential targets to constrain the aggressive malignancy seen in the castration-resistant PCa.

## Introduction

Prostate cancer (PCa) is the most commonly diagnosed cancer and the second leading cause of cancer-related deaths among adult males globally [1]. In recent years, its incidence has been progressively increasing [2], making it a detrimental public health problem worldwide. During PCa development, androgen receptor (AR) acts as the primary mediator of growth and survival of cancer cells through different androgen-mediated mechanisms, which is the basis for the front-line treatment with androgen-deprivation therapy (ADT). However, within few years approximately 20% of cases progress to a metastatic and androgen-independent stage of the disease known as castration-resistant prostate cancer (CRPC) [3]. Resistance develops mainly through overexpression and amplification of the androgen receptor or through mutations of the receptor to a constitutively active form [3]. Metastatic castration-resistant prostate cancer (mCPRC) is associated with a poor prognosis and mean survival time of only 16–18 months [4]; moreover, approved therapies work only to alleviate symptoms and prolong life, making mCRPC an incurable disease to this date [5]. Thus, understanding the mechanisms that underlie the pathogenesis of mCRPC is fundamental for defining appropriate novel therapeutic targets for this disease.

Metastatic CRPC is thought to be initiated in part by a mechanism called epithelial-to-mesenchymal transition (EMT) characterized by morphological changes of PCa cells from cuboidal to spindle-like shapes [6, 7]. Concomitantly, epithelial markers like E-cadherin and cytokeratins get downregulated leading to loss of cell-to-cell and cell-to-extracellular matrix adhesions, whereas mesenchymal markers such as vimentin and N-cadherin are upregulated prompting enhanced cell mobility, invasion, and metastasis [6–8]. EMT plays a critical role in cancer progression and metastasis; however, the exact mechanism of how cancer cells undergo EMT is still not clearly understood [9].

Besides EMT, strong evidence has emerged along the years pointing to the major contributing role of inflammation in PCa initiation and progression [10–12]. In fact, histological examination of prostate tissues of men with PCa reveals a microenvironment rich in inflammatory cells [13]. Many studies in the field of PCa also focused on scrutinizing the function of genetic polymorphism induction of inflammatory pathways and production of cytokines in this context [12]. Of all the cytokines that have been explored and correlated with PCa, interleukin-6 (IL-6) has been widely investigated and recognized as a major regulator and influencer in the progression of PCa [11]. IL-6 acts through activating different signaling pathways upon binding with IL-6 receptor, including the stress-activated protein kinase 2 (SAPK2/p38)-mitogen-activated protein kinase (MAPK) pathway [11].

The p38-MAPK, in particular, is usually activated by environmental stresses (osmotic stress, oxidative stress, heat), in addition to proinflammatory cytokines (TNFα, IL-6 or IL-1), anti-inflammatory cytokines, and transformation growth factors (EGF, TGF-β) [14, 15]. Once activated, p-p38 controls numerous transcriptional factors such as NF-κB, ELK-1, MEF-2, MAC, p53, and STAT1 [16–18], as well as some cell cycle and apoptotic mediators such as BCL-2 [19]. Additionally, p38 boosts survival of cells under stress stimuli such as DNA damage [15, 19]. Accordingly, numerous studies verified the substantial role of p38 in several cancers mainly leukemia [20], lymphoma [21], breast cancer [17], gastric cancer [22], lung cancer [23], and to our interest PCa [24]. Scientists revealed that p-p38 is overexpressed in prostatic intraepithelial neoplasia (PIN), as well as in well- and moderately-differentiated cancers [15]. Nevertheless, the role of p38 has been controversial where it has been described as proapoptotic in several PCa *in vitro* models [25–30], yet contributes to PCa progression via promoting tumor growth, androgen independence, and metastasis [15]. In accordance with what has been previously mentioned, IL-6 induces resistance to therapy through the p38-MAPK pathway [31].

Our knowledge of the molecular players and inflammatory cytokines that contribute to the progression of PCa to an advanced stage is very lagging. Therefore, further efforts are warranted to decipher the role of such mediators in the progression of the disease from primary stages to CRPC [32, 33], taking into consideration well-established *in vitro* models of primary vs. advanced PCa. In our previous study, we developed novel murine PCa cell lines that represent the sequence of androgen dependent (AD)-to-androgen independent (AI) PCa progression [8], suggesting that these can serve as viable models to survey PCa progression. Henceforth, in the present study we aimed to identify novel potential biomarkers, therapeutic targets and biological pathways pertaining to PCa progression. We identified functional and evolutionarily conserved gene expression programs in the progression of PCa employing the novel murine PCa models PLum-AD and PLum-AI. Then, we used these functional gene expression profiles to identify and delineate potential targets, mainly IL-6 and p38-MAPK, which we validated phenotypically at the molecular and functional levels.

## Materials and methods

### Microarray and functional pathway analysis

Microarray data analysis was performed within R statistical language and environment. Raw data was previously normalized using the Robust Multiarray Averaging (RMA) method [8, 34]. Data representing the transcriptomes of PLum-AD and PLum-AI cells (n = 3) was analyzed using BRB Array tools v.3.7.0 [35]. Identification of gene features significantly differentially expressed between PLum-AD and PLum-AI cells was performed in the R statistical language and environment using a false discovery rate (FDR) threshold of 10% and based on a double threshold of adjusted p-value less than 0.001 and a logged fold-change threshold of at least 2 was further applied. Differentially expressed gene features were then functionally analyzed and topologically organized into gene-gene interaction networks using the commercially available software Ingenuity Pathways Analysis (IPA) as previously performed [36, 37]. The latter shaped gene features into molecular gene-sets and pathways significantly modulated between PLum-AI and PLum-AD adenocarcinoma cells. Transcripts were also analyzed by gene set enrichment analysis (GSEA) using IPA which aided in identifying upstream regulators of the transcript profiles. Gene network analysis allowed us to examine functional associations among the genes and generate gene networks with high significance based on the presence of a higher number of inter-connected genes than expected by chance. This helped us in identifying network hub genes predicted to have key roles based on their expression and number of functional associations and pursuing the validation of those gene.

### Cell culture

#### Cell lines

Our group has previously generated a novel murine *in vitro* model recapitulating PCa progression from a primary androgen-dependent state (PLum-AD cells), to an advanced androgen-independent state (PLum-AI cells) [8, 38]. Molecular, functional and pathophysiological characterization of both murine cell lines was formerly observed [8] that PLum-AD cells, indeed, represent primary PCa while PLum-AI cells display an aggressive nature and thus represent the advanced stages of the disease. Yet, it is important to note that both cells carry the same genetic background (*Pten*^-/-^ *TP53*^-/-^) [8, 38].

#### 2D Culture

PLum-AD and PLum-AI cells were cultured and maintained in Advanced DMEM/F-12 (Dulbecco's Modified Eagle Medium/Ham's F-12) medium (Gibco) supplemented with 1% Penicillin/Streptomycin (Lonza), 0.2% Plasmocin™ prophylactic (Invivogen), 1.5% Gentamicin/Amphotericin (Invitrogen), 1% Hepes (Gibco), 1% Glutamax (Gibco) and 10 ng/ml Epidermal Growth Factor (EGF) (R&D Systems). Cells were incubated at 37°C in a humidified 5% CO_2_ incubator.

#### Preparation of treatments

The p38 MAPK inhibitor SB203580 (Abcam) was reconstituted in 0.1% dimethylsulfoxide (DMSO), at a concentration of 10 μM, aliquoted, and then stored at -20°C. Recombinant human IL-6 (Eurobio) was used at a concentration of 10 ng/mL in Phosphate Buffered Saline (PBS) (Sigma-Aldrich). The concentrations of both treatments used was determined based on previous reports from the literature [39, 40].

### Total RNA extraction and quantitative real-time polymerase chain reaction (qRT-PCR)

We sought to validate the effect of SB203580 and IL-6 treatments on gene expression of PLum-AI and PLum-AD respectively. Therefore, we performed qRT-PCR on select genes that were differentially expressed between PLum-AI and PLum-AD based on the microarray data analysis and subsequent gene network analyses. Accordingly, we analyzed the expression of a total of 10 murine genes (S1 Table).

PLum-AI and PLum-AD cells (n = 3) were treated with SB203580 and IL-6 respectively, compared to a control condition (media without treatment), and collected after 72 hours. Total RNA was extracted with the RNeasy Plus Mini Kit (Qiagen) and cDNA was then synthesized using the QuantiTect Reverse Transcription Kit (Qiagen) according to the manufacturers’ protocols. The qRT-PCR reaction was carried out with a homemade 2X buffer using the BioRad CFX96 RT-PCR detection system. Relative gene expression was analyzed using the 2^-ΔΔCt^ method and normalized against the expression of Glyceraldehyde Phosphate Dehydrogenase (*Gapdh*) as reference gene. The primers used in PCR (shown in S1 Table) were designed using Primer-Blast and synthesized at Macrogen. All reactions were carried out in duplicates.

### Immunofluorescence and confocal microscopy

Antibodies from the indicated manufacturers used in this study were as follows: mouse monoclonal anti-CK8 (1/200 dilution) (Covance); rabbit polyclonal anti-vimentin (1/200 dilution) (Santa Cruz Biotechnology); Alexa 488 goat anti-rabbit, and Alexa 568 goat anti-mouse (Invitrogen). Secondary Alexa Fluor antibodies were used at 1/200 dilution. Fluoro gel II with Dapi was purchased from EMS (Electron Microscopy Sciences).

Prostate epithelial lineage markers expressed by the PLum-AD and PLum-AI cells grown in 2D monolayer cultures were characterized using indirect immunofluorescence analysis. Adherent cells, grown on cover slips, from the three different conditions (control, treatment with SB203580, and treatment with IL-6) were fixed using 4% paraformaldehyde (PFA) in PBS for 10 minutes followed by permeabilization with 0.5% Triton X 100 in PBS for 4 minutes. The cells were then incubated in blocking buffer [0.1% BSA, 0.2% Triton X 100, 0.05% Tween 20, and 10% normal goat serum (NGS) in PBS] for half an hour to block non-specific sites. Afterwards, cells were incubated overnight at 4˚C with the specified primary antibody in 2% BSA in PBS. The next day, cells were washed three times with PBS containing 0.1% Tween 20 and incubated, in the dark, with Alexa 488 and/or 568 conjugated IgG secondary antibodies in 2% BSA in PBS for 30 minutes at room temperature. Lastly, cells were washed and mounted using anti fade reagent (Fluoro gel II with Dapi).

Using the 40× objective on a Carl Zeiss LSM 710 laser scanning confocal microscope, manual quantification of the percentage of cells expressing double positive cytokeratin 8/vimentin (CK8/Vim), Vim alone or CK8 alone, from the total number of cells counted, was performed. Positively-stained cells from at least ten different representative fields chosen from three independent experiments were counted and numbers were plotted as percentages out of the total number of positively stained cells per experiment. Confocal microscopic analyses were performed using the Carl Zeiss ZEN 2012 image software.

### MTT cell proliferation assay

Cellular proliferation of PLum-AD and PLum-AI upon treatment with SB203580 or IL-6 was assessed using the 3-(4,5-dimethylthiazol-2-yl)-2,5-diphenyltetrazolium bromide (MTT) assay. Cells were seeded in 96-well plates in triplicates and treated with SB203580 or IL-6 for 24, 48 and 72 hours. At each time point, cells were incubated with 10 μL of 5 mg/mL (in 1x PBS) MTT dye at 37°C, 5% CO_2_ for 3 hours. Afterwards, media was aspirated and 100 μL of isopropanol was added to each well and incubated for at 37°C 15 minutes to solubilize the formazan crystals. Absorbance was measured on a Multiskan EX ELISA Reader at 595 nm. Cell proliferation was calculated using the percentage of the optical density (OD) ratio of treated cells relative to control (media without treatment). Data represents the average of three experiments.

### Wound healing cell migration assay

PLum-AD and PLum-AI cells were plated at a density of 250,000 cells/well in 1 mL complete growth medium in 12-well culture plates and incubated at 37°C in a humidified 5% CO_2_ incubator overnight until 80–90% confluency. In the case of PLum-AI cells, wells were coated with 1% type I collagen prior to seeding to prevent cell detachment. Cells were then treated with 10 μg/mL of Mitomycin C (Sigma-Aldrich) for 2 hours for PLum-AD and 0.5 μg/mL for 3 minutes for PLum-AI at 37°C to block cellular proliferation and to assure that closure of the wound is a function of cellular migration and not cellular proliferation. Two uniform scratches of nearly the same width were made in each well using a 200 μL micropipette tip, and the plates were then washed twice with PBS to remove the detached cells. Remaining cells were cultured in complete growth media with or without treatment for 48 hours. Photos were taken at 0 and 48 hours, and the distance travelled by the cells was computed from the closure of the wounds and expressed as percentage of wound closure with each treatment compared to the control condition. Data obtained from this experiment represents an average of three independent experiments, each in duplicates.

### Trans-well cell migration assay

Cells were starved overnight the day before the experiment. Each cell line was seeded at a density of 2 × 10^5^ cells/well for 3 different conditions onto 8 μm pore size inserts (Falcon) which were placed in 24 well plates containing media without FBS. The lower chamber was filled with media containing 5% FBS as chemoattractant. The cells were then incubated at 37°C in a humidified 5% CO_2_ incubator. After 72 hours, the inserts were removed, and cells that failed to migrate through the inserts were smoothly scraped off using a cotton swab. Cells on the inserts were fixed in 4% PFA and stained with hematoxylin and eosin (H&E). The membrane of the inserts was then cut and mounted on a microscopic slide for examination using a light microscope. Migration quantification was performed by counting the number of cells that migrated through the inserts. Six different randomly selected fields were photographed and counted under 10× magnification of an inverted light microscope. Data obtained from this experiment represent an average of three independent experiments.

### Sphere formation assay

Sphere formation assay was performed as previously described by our group (Bahmad *et al*., 2018) [41]. In brief, single cells were suspended, in duplicates, in a 50 μL volume of 1:1 cold growth factor-reduced Matrigel™ (BD Biosciences)/growth medium at a density of 2,000 cells/well. This cell suspension was plated gently around the rim of each well of a 24-well plate and left for 1 hour at 37°C in a 5% CO_2_ humidified incubator to solidify. Afterwards, 500 μL of Advanced DMEM/F-12 cell growth medium supplemented with 5 ng/mL EGF with or without treatment with either SB203580 or IL-6 was gently added to the center of each well and replenished every 2–3 days. Prostatospheres were harvested at 12–15 days after plating and their count is assessed using the sphere formation efficiency or sphere formation unit (SFU) formula: SFU (in %) = (number of spheres counted ÷ number of input cells) × 100. Average diameter of prostatospheres was also evaluated for the different conditions (average of 30 spheres per condition from three independent experiments) and Zeiss Axiovert microscope was used for the acquisition of bright field images of the cultured prostatospheres.

### Statistical analyses

Statistical analysis was performed using GraphPad Prism 8.0 Software and the R system was used for statistical genomic data computing. Data included herein represents the means ± SEM of three independent experiments as noted in the figure legends. P-values were computed using one-way ANOVA statistical test, followed by multiple comparisons using Bonferroni post hoc analysis in IF quantifications, MTT assay, and trans-well migration assay. Two-tailed unpaired Student’s t-test was used to compare between the control and each treatment in PCR analysis, wound-healing assay and sphere formation assay. Statistical significance was reported when the P-value was less than 0.05 (*P<0.05; **P<0.01; ***P<0.001).

## Results

### Gene expression profiles are differentially expressed between PLum-AI and PLum-AD cells

We used cell lines that were previously generated from parental PLum cells in androgen-rich and androgen-depleted environments, namely PLum-AD (androgen-dependent) and PLum-AI (androgen-independent) murine cell models respectively, to recapitulate PCa progression [8, 38]. To understand the mechanisms underlying this progression, we first sought to compare global gene expression profiles of PLum-AI and PLum-AD cells in order to create a holistic picture of the differentially altered molecular signatures between the two, i.e. identify differentially expressed gene features that might contribute to PCa progression.

Microarray hybridization was performed on the mouse Affymetrix Gene Chip 2.0 ST as previously reported [8]. The data discussed in this paper have been deposited in NCBI's Gene Expression Omnibus [42] and are accessible through GEO Series accession number GSE151187 (https://www.ncbi.nlm.nih.gov/geo/query/acc.cgi?acc=GSE151187). Differentially expressed genes were defined based on the criteria of p-value < 0.001 of the univariate *t*-test with permutation and estimation of false-discovery rate (FDR) of 10% and at least 2-fold change in expression [37]. Gene expression profiling revealed 723 differentially expressed genes, 392 of which were upregulated and 331 were downregulated in PLum-AI compared to PLum-AD cells. Significantly activated or inhibited (predicted and indicated by a computed z-score) upstream regulators of the identified differentially expressed transcripts were interrogated using the GSEA feature by IPA^®^ software. Representative upstream regulators that were either upregulated or downregulated in PLum-AI versus PLum-AD cells are shown in S2 and S3 Tables (all *p* < 0.0001). These included inhibition of Sirolimus (Rapamycin) (z-score = -2.461) and PRKAA2 (z-score = -2.829) and marked activation of IL-6 (z-score = 3.791), EGF (z-score = 2.491), and MAPK1 (z-score = 2.469).

Interestingly, GSEA showed that gene sets previously shown to be associated with favorable response to the p38 inhibitor SB203580 (z-score = -3.461) were attenuated (inversely enriched) in PLum-AI cells suggesting that these aggressive cells may be therapeutically vulnerable to p38 inhibition. Further functional analysis using IPA emphasized the significant modulation of other signaling networks, particularly those associated with enhanced EMT, inflammation and immune function/response such as *Tnf*, *IL-6*, *Mmp13*, *Twist*, and *Ccl2* (all *p* < 0.001). Our GSEA confirms the purity of the cell lines used recapitulating PCa progression.

**Fig 1:**
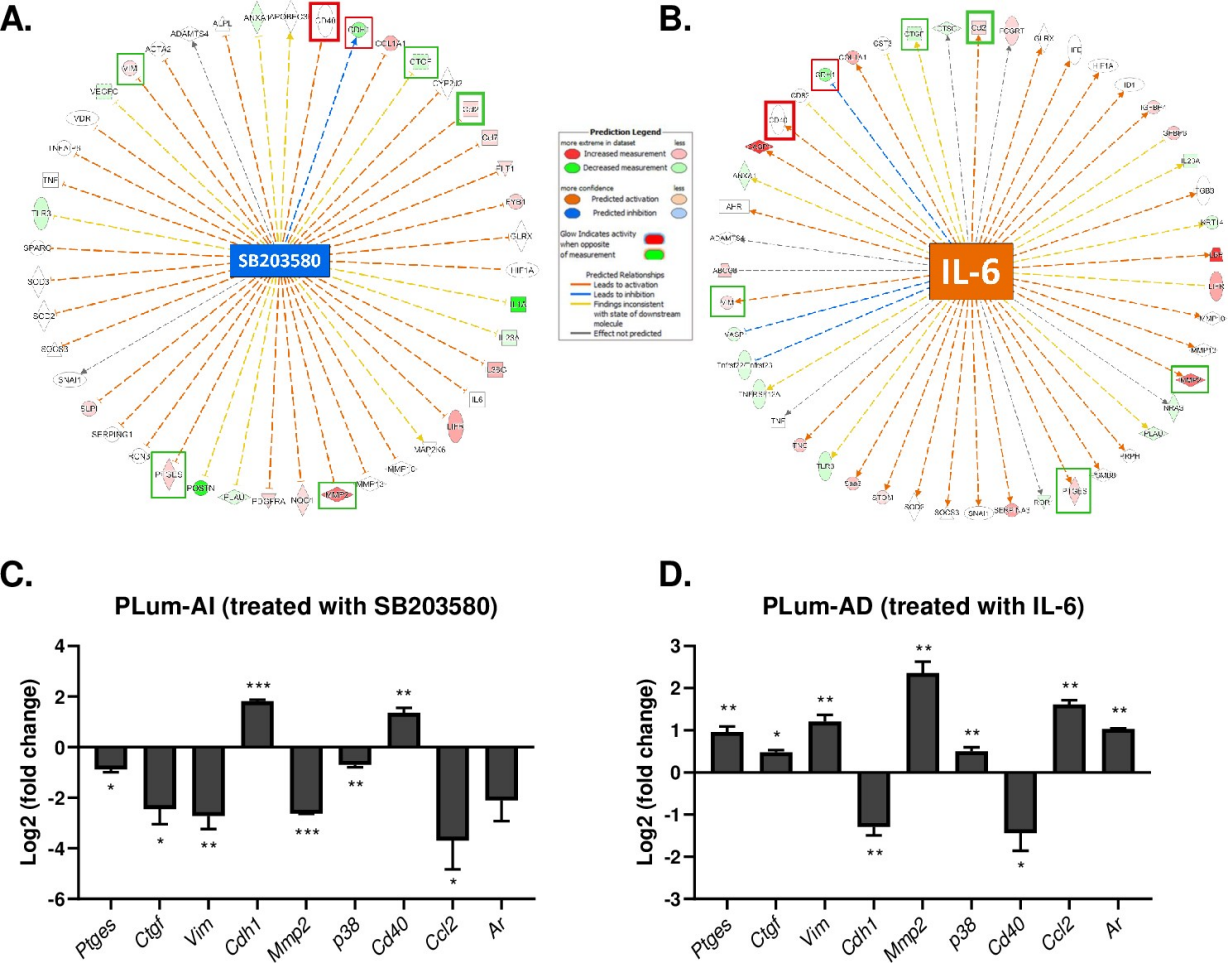
Gene network analysis of differentially expressed genes around **(A)** SB203580 and **(B)** IL-6 in PLum-AI vs. PLum-AD and validation of representative genes by qRT-PCR. Significantly upregulated and downregulated genes were identified around **(A)** SB203580 and **(B)** IL-6. Genes are represented as nodes, and biological relationships between the center and the other nodes are represented as an edge (flat line). The intensity of the node color indicates the degree of upregulation or downregulation. Red boxes signify overexpressed genes in primary and localized PCa, and green boxes indicate overexpressed genes in advanced and metastatic CRPC. **(C and D)** PLum-AI and PLum-AD cell lines were treated with SB203580 (10 μM) and IL-6 (10 ng/mL) respectively, and relative expression of a number of differentially expressed genes was determined. **(C)** *p38*, *Mmp2*, *Ptges*, *Ctgf*, *Vim*, *Ar* and *Ccl2* were significantly downregulated in PLum-AI cells after treatment with SB203580 and significantly upregulated in PLum-AD cells after treatment with IL-6. **(D)** Conversely, *Cdh1* and *Cd40* were significantly upregulated in PLum-AI cells after treatment with SB203580 and significantly downregulated in PLum-AD cells after treatment with IL-6. The relative expression of the genes of interest was normalized to the housekeeping gene *Gapdh*. The data are reported as mean ± SEM (*P < 0.05, **P < 0.01 and ***P<0.001; every treated cell line was compared to its control, Student’s *t*-test).

Functional pathway and gene-network analysis using IPA^®^ software showed that expression levels of several *Mmp* genes such as *Mmp2*, *Mmp10* and *Mmp13*, as well as *Tnf*, connective tissue growth factor (*Ctgf*), Prostaglandin E synthase (*Ptges*), and *Vim* would be vulnerable to inhibition through the p38 MAPK pathway by SB203580 (Fig 1A). On the other hand, gene-network analysis of IL-6 showed some common genes with the gene-network analysis of SB203580, but on the contrary suggesting their activation rather than inhibition (Fig 1B). Indicated by red or green boxes in Fig 1A and 1B are common genes that were validated using qRT-PCR, with red signifying overexpressed genes in primary and localized PCa, and green indicating overexpressed genes in advanced and metastatic CRPC.

### Differentially expressed genes between PLum-AI and PLum-AD in selected functional groups were validated using qRT-PCR

As previously discussed, we sought to validate some of the common genes from the gene-network analysis of both SB203580 and IL-6. Therefore, we investigated the effect of SB203580 and IL-6 on gene expression of PLum-AI and PLum-AD cells respectively. Based on the gene set analysis, the two treatments are predicted to exert opposing effects on these cells. SB203580 will trigger mesenchymal-to-epithelial reverting transition (MErT) of mesenchymal PLum-AI cells, and IL-6 will induce EMT of epithelial PLum-AD cells.

On one hand, gene-network analysis of SB203580 revealed the downregulation of numerous genes fostering EMT and the upregulation of genes characterized in epithelial phenotypes in PLum-AI cells (Fig 1A). Gene-network analysis of IL-6, on the other hand, suggested the upregulation of genes enhancing EMT and downregulation of some epithelial markers (Fig 1B). For this reason, we selected some common genes between the two networks to validate using qRT-PCR. Genes that were predicted to be affected by both SB203580 and IL-6 included *p38*, *Ctgf*, *Ptges*, *Mmp2*, *Vim*, *Cdh1*, *Ccl2* and *Ar*. All these genes, except for *Cdh1* which is an epithelial cadherin protein-coding gene and *Cd40*, a member of the TNF receptor superfamily and often expressed on epithelial malignancies [43], are known to be pivotal to the tumor microenvironment and are indeed widely expressed in progressive cancers [44–47].

Consistent with the gene set analysis results, treatment of PLum-AI cells with SB203580 induced a significant downregulation in *p38*, *Mmp2*, *Ptges*, *Ctgf*, *Vim*, *Ccl2* and *Ar* expressions, whereas IL-6 induced the opposite effect in the expressions of these genes in PLum-AD cells. Additionally, as predicted by the enrichment analysis, a statistically significant upregulation in the expressions of *Cdh1* and *Cd40* was recorded in PLum-AI cells treated with SB203580, and a significant downregulation in PLum-AD cells when exposed to IL-6 (Fig 1C and 1D).

### Immunofluorescence characterization of PLum-AD and PLum-AI cells upon treatment with SB203580 or IL-6 validated the MErT effect of the former and the EMT effect of the latter

To further confirm the effect of SB203580 (10 μM) and IL-6 (10 ng/mL) on PLum-AD and PLum-AI cells at the molecular and morphological levels, we performed immunofluorescence characterization and quantification of two EMT markers, Cytokeratin (CK)-8 (an epithelial luminal cell marker) and Vimentin (Vim) (a mesenchymal cell marker).

**Fig 2:**
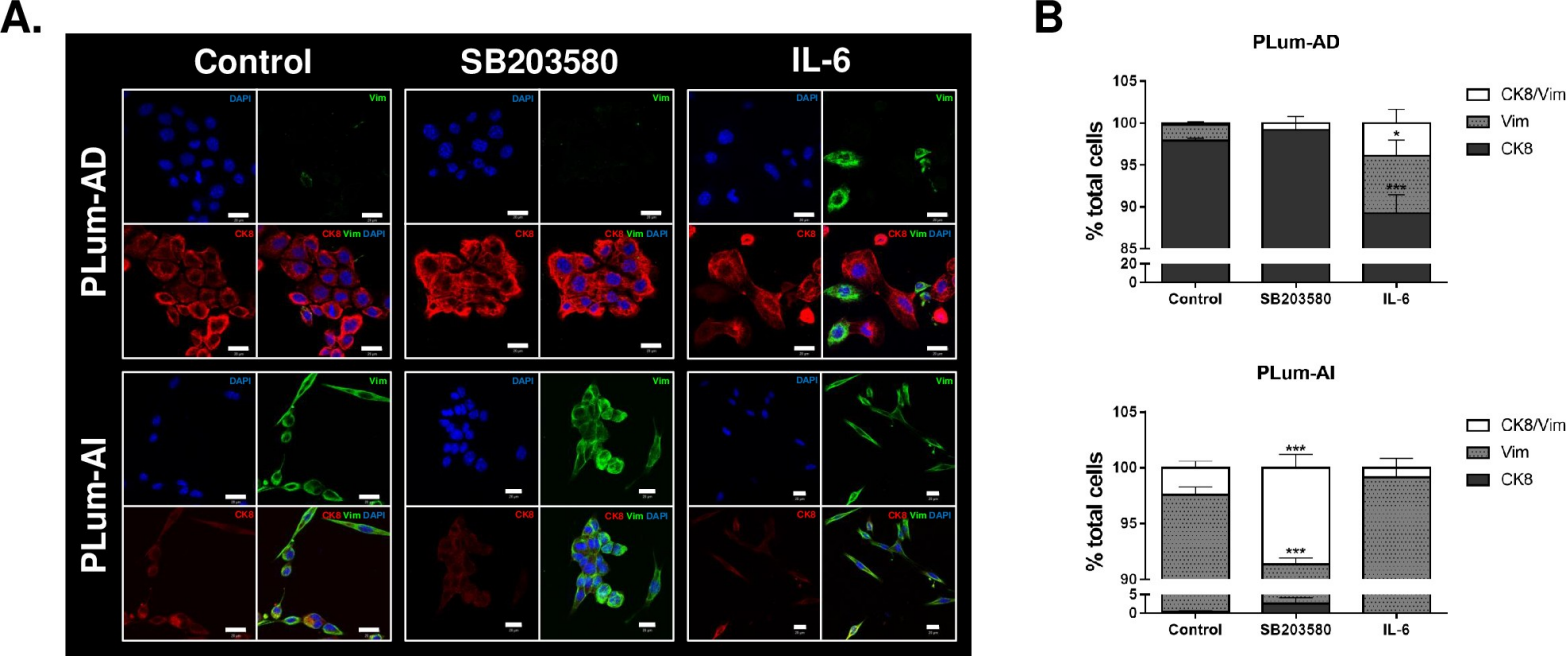
Immunofluorescence characterization of PLum-AD and PLum-AI cells upon treatment with SB203580 (10 μM) and IL-6 (10 ng/mL). **(A)** Representative immunofluorescent images of PLum-AD and PLum-AI cells stained for EMT markers (epithelial CK8 and mesenchymal VIM) and the nuclear counterstain DAPI are shown. Scale bars = 20 μm. **(B)** Quantification of the percentage of cell counts expressing double positive CK8/Vim, Vim alone or CK8 alone. Data are expressed as the percentage of positively stained cells for each marker with respect to the total number of cells. Data represent an average of three independent experiments and are reported as mean ± SEM (*p<0*.*001*, Two-way ANOVA; *P<0.*05*; ***P<0.*001*; different treatment conditions compared to the control for each marker and for each cell line, Bonferroni’s multiple comparison’s test).

In PLum-AD cells, while the untreated cells primarily expressed CK8 with an average of 97.89 ± 0.32%, the expression of CK8 increased to 99.19 ± 0.81% upon treatment with SB203580 and decreased tremendously and significantly to 89.23 ± 2.22% (p-value < 0.001) when treated with IL-6. On the contrary, quantification of Vim expression revealed a significant increase from 1.93 ± 0.20% to 6.87 ± 1.84% in IL-6-treated PLum-AD cells compared to their control (p-value < 0.05). Interestingly, treating cells will SB203580 completely abolished Vim expression (Fig 2B, top panel). These data suggest that IL-6, per se, induces a change in the morphology of PLum-AD cells from an epithelial (CK8) to a mesenchymal (Vim) phenotype, hence indicating an EMT process.

Quantification of the expression levels of the two previously selected EMT markers in PLum-AI cells upon treatment with either SB203580 or IL-6 demonstrated a statistically significant difference in the SB203580-treated group compared to its control. In this group, a considerable decrease in Vim expression from 97.19 ± 0.72% to 88.76 ± 0.56% was recorded after treatment (p-value < 0.001). Besides, the percentage of PLum-AI cells expressing double-positive CK8/Vim staining increased significantly from 2.43 ± 0.60% in the control group to 8.65 ± 1.19% when treated with SB203580. As for IL-6-treated cells, the percentage of PLum-AI cells expressing Vim was maintained after treatment at 99.17 ± 0.83% compared to 97.19 ± 0.72% in the control, and this was noteworthy accompanied by a decrease in the percentage of CK8/Vim double-positive expressing cells from 2.43 ± 0.60% in the control group to 0.83 ± 0.83% in the treatment group (Fig 2B, lower panel). Our results confirm that treating PLum-AI cells with SB203580 promotes a MErT process and a reversal of PCa progression via change in the morphology of cells from a mesenchymal (Vim) to an epithelial (CK8) phenotype.

### SB203580 reduces while IL-6 promotes both PLum-AD and PLum-AI cell proliferation in a time-dependent manner

**Fig 3:**
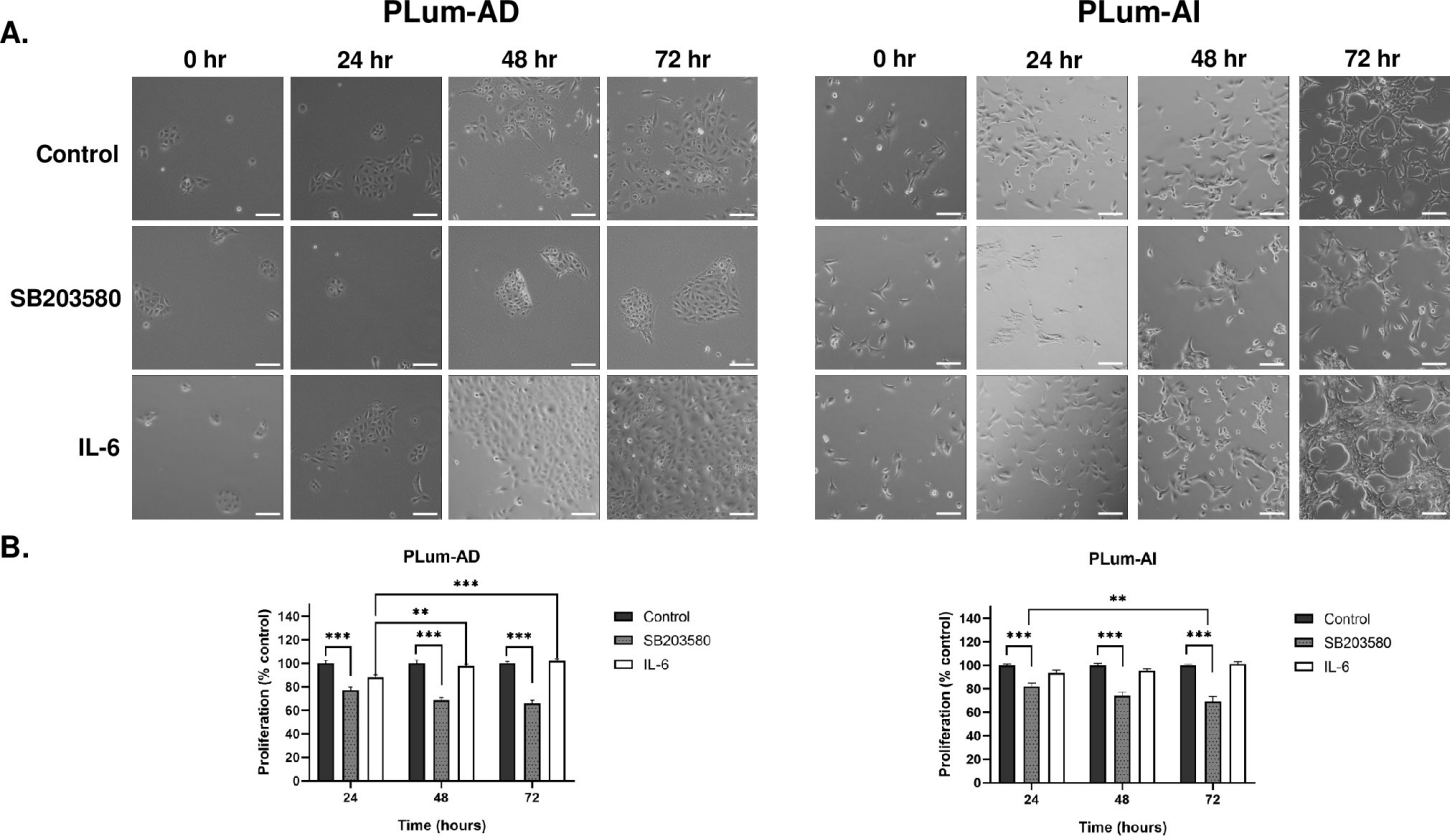
SB203580 reduces while IL-6 promotes the proliferation of Plum-AD and PLum-AI cells in a time-dependent manner. **(A)** Representative bright-field images of PLum-AD and PLum-AI cells showing effect of treatment with SB203580 (10 μM) or IL-6 (10 ng/mL) on cell proliferation and morphology. Scale = 100μm. After incubation of cells for 24, 48 and 72 hours with or without treatment with SB203580 or IL-6, cell proliferation was determined using MTT assay. **(B)** Results are expressed as percentage of proliferation of the treated group compared to control at every time point. Data represents an average of three independent experiments and is reported as mean ± SEM (Two-way ANOVA with Bonferroni’s corrected test for multiple comparisons *P < 0.05; **P < 0.01; ***P < 0.001).

Next, we sought to assess the *in vitro* anti-proliferative effect of SB203580 and the pro-proliferative effect of IL-6 on both PLum-AD and Plum-AI cells via MTT assay (Fig 3). Fig 3A shows the morphological and confluency changes after treatment with SB203580 and IL-6 in both PLum-AD and PLum-AI, compared to the control in each. Treatment with IL-6 prompted PLum-AD cells to undergo EMT thus demonstrating a mesenchymal morphology (migratory phenotype with minimal cell-cell interactions). On the contrary, treatment with SB203580 had the opposite effect on PLum-AI cells that reverted to an epithelial morphology (cobble-stone appearing cells with well-defined cell-cell interactions) (Fig 3A).

Our results demonstrated that SB203580 had statistically significant anti-proliferative effects on both PLum-AD and PLum-AI cells, whereas IL-6 displayed a pro-proliferative effect on both cells after 3 consecutive days of treatment. In PLum-AD cells, treatment with SB203580 reduced proliferation to 65.69 ± 2.72% after 72 hours (p-value < 0.001), while treatment with IL-6 increased cell proliferation to 102.22 ± 1.56% at 72 hours (Fig 3B). In PLum-AI, the effect of SB20350 was also evident where proliferation of cells significantly decreased to 69.26 ± 4.16% after 72 hours of treatment (p-value < 0.001), whereas IL-6 increased cell proliferation to 101.37 ± 1.86% after 72 hours (Fig 3B).

The anti-proliferative effect of SB203580 and the pro-proliferative effect of IL-6 on both cell lines further confirmed our GSEA that revealed the sensitivity to these treatments. Although the effect of IL-6 was not statistically significant in PLum-AD cells, however the trend was noticeable pointing to its effect.

### SB203580 reduces while IL-6 promotes both PLum-AD and PLum-AI cell migration

**Fig 4:**
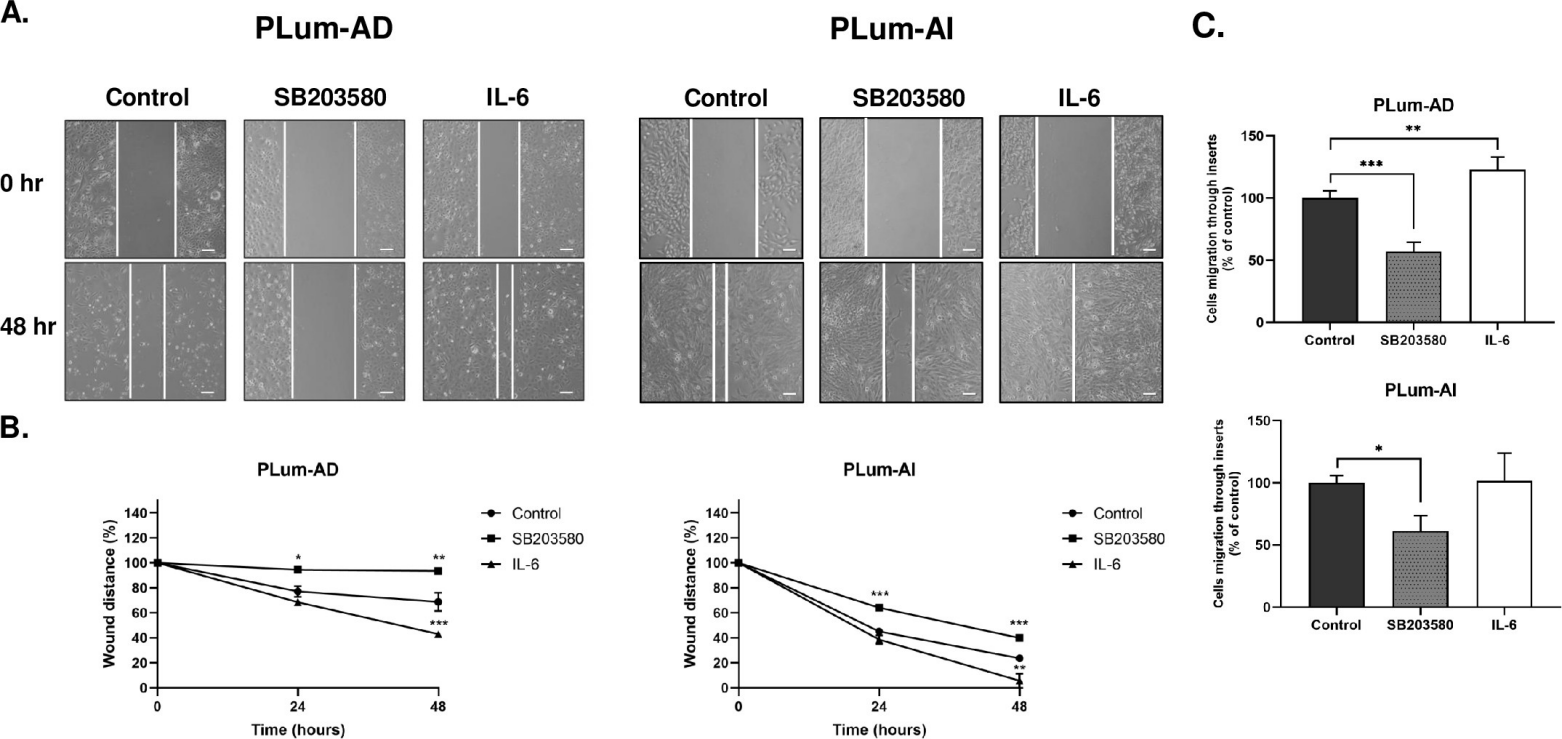
SB203580 reduces while IL-6 promotes PLum-AD and PLum-AI migratory potential in a time-dependent manner. **(A)** Representative bright-field images showing effect of treatment with SB203580 (10 μM) and IL-6 (10 ng/mL) on cell migration. Scale = 100μm. A scratch was made in a 12-well plate of confluent PLum-AD or Plum-AI cells using a 200 μL tip, and images were taken at T = 0 and 48 hours. **(B)** Quantification of the distance of wound closure (wound distance in %) was assessed over time and represented by the line graphs. **(C)** Trans-well migration assay. PLum-AD and PLum-AI cells were plated onto 8 μm pore size inserts placed in 24-well plates containing media with or without treatment with SB203580 or IL-6. Media containing 5% FBS was used in the lower chamber as chemoattractant. Quantification of the migrated cells was assessed by taking the average of migrating cells counted in 6 randomly selected fields for every condition at 10x magnification of an inverted light microscope. Data represents an average of three independent experiments and are reported as mean ± SEM (One-way ANOVA with Bonferroni’s corrected test for multiple comparisons of each treatment with the control *P < 0.05; **P < 0.01; ***P < 0.001).

Knowing that primary localized PCa can turn aggressive and develop the potential to metastasize to distant body organs such as bones, brain, lymph nodes, liver, and thorax [48], we pursued confirmation of this hallmark via investigating the effect of both SB203580 and IL-6 on the migratory abilities of our two murine PCa cell models. This validation will further confirm our GSEA predictions that SB203580 is associated with inhibition of genes contributing to metastasis, such as *Tnf*, *Vim* and the *Mmp* family, while IL-6 manifests an opposite effect. To test this hypothesis, two assays were performed: the wound healing and trans-well cell migration assays (Fig 4).

In the wound healing assay, PLum-AD and PLum-AI cells were treated with 10 μM of SB203580 or 10 ng/mL of IL-6 for 48 hours. Representative bright-field images of wound closure in the three different conditions at start and termination of experiments are shown in Fig 4A. Our results revealed that while wound closure of PLum-AD cells in the control group reached 32%, wound healing was significantly suppressed by SB203580 treatment after 48 hours where the wound was able to close by only 6% (p-value < 0.01), whereas IL-6 significantly promoted this migration where wound closure reached nearly 57% after 48 hours of incubation (p-value < 0.001) (Fig 4B). Similarly, for PLum-AI cells, wound healing was significantly reduced from 76% in the control group to 60% upon treatment with SB203580 (p-value < 0.001), but significantly increased to 94% with IL-6 (p-value < 0.01) (Fig 4B).

Consistent with the wound-healing assay results, SB203580 significantly decreased the migratory ability of PLum-AD cells to 57% compared to the control (p-value < 0.001) as assessed by the trans-well cell migration assay. Treatment with IL-6, on the other hand, presented a statistically significant enhancement in the migratory capacity of PLum-AD cells, with this increase reaching nearly 123% compared to the control (p-value < 0.01) (Fig 4C).

As for PLum-AI cells, their migratory potential was also significantly reduced by SB203580 to 61% compared to the control, after 72 hours (p-value < 0.05), whereas IL-6 had no significant increase in this potential where it was maintained at 101% (Fig 4C). Our results here confirm that IL-6 augments the migratory capabilities of PLum-AD and PLum-AI cells, which is a vital hallmark in promoting PCa progression to a more advanced and metastatic stage, while SB203580 exerts the opposite effect by suppressing the migration potential in these cells.

### SB203580 decreases while IL-6 augments the stem/progenitor cell properties of PLum-AD and PLum-AI *in vitro*

**Fig 5:**
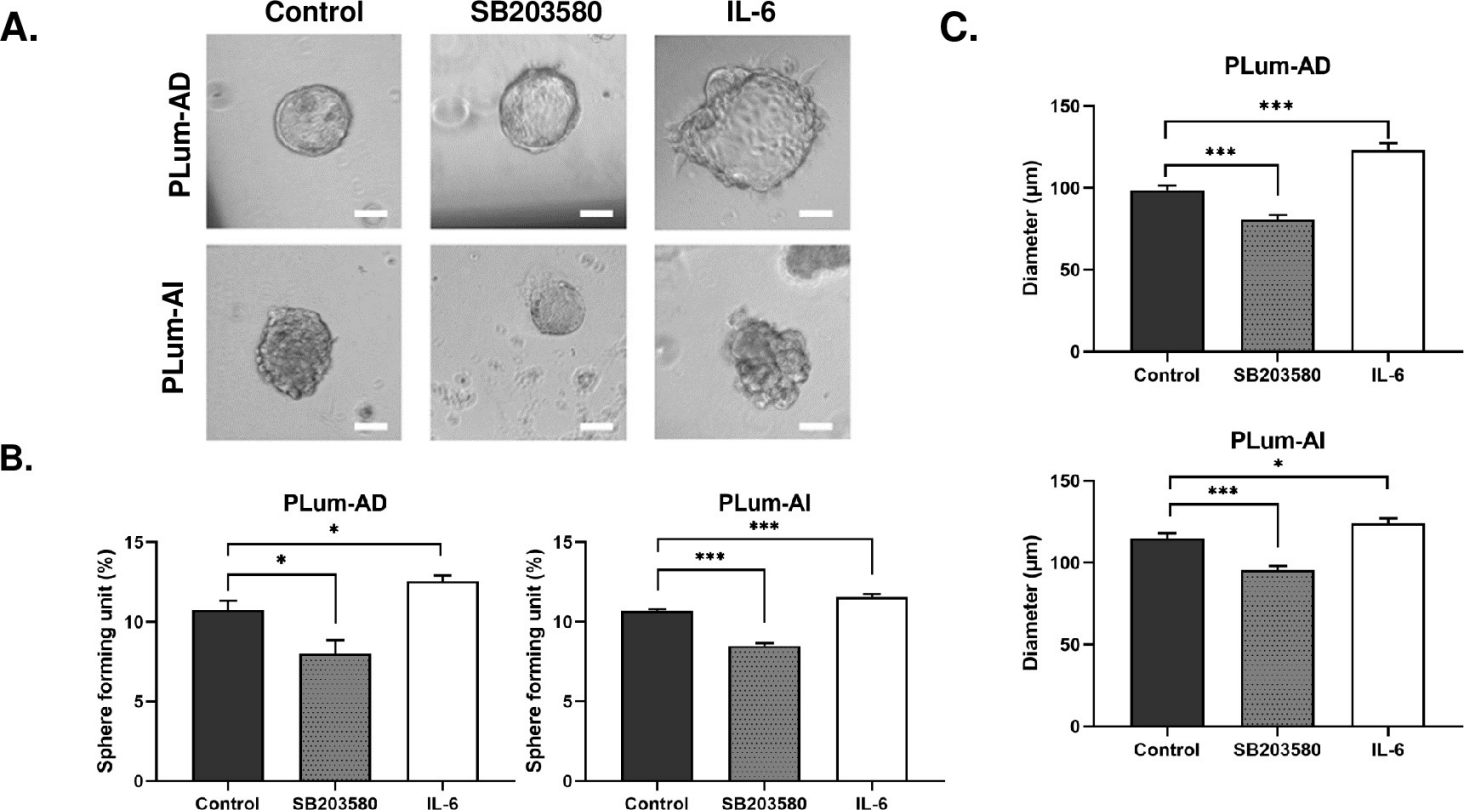
SB203580 reduces while IL-6 augments the sphere forming units and diameter of PLum-AD and PLum-AI prostatospheres. **(A)** Representative bright-field images of PLum-AD and PLum-AI prostatospheres in different conditions embedded in Matrigel^TM^ at day 12 after plating. Scale bar = 50 μm. Images were visualized by Axiovert inverted microscope and analyzed by Carl Zeiss Zen 2012 image software. Prostatospheres formed in each well were counted at day 12 for both cell lines. **(B)** Treatment with SB203580 (10 μM) significantly decreased sphere forming units (SFUs) while IL-6 (10 ng/mL) increased it in both cell lines. **(C)** SB203580 significantly reduced the average diameter of spheres while IL-6 increased it in PLum-AD and PLum AI. Data represents the average diameter (μm) of 90 prostatospheres in each condition from three independent experiments. All data is reported as mean ± SEM (*P < 0.05, **P < 0.01 and ***P<0.001; every treatment condition was compared to its control, Student’s t-test).

Finally, we sought to assess the potential effects of SB203580 and IL-6 on the cancer stem cell (CSC) population of PLum-AD and PLum-AI. Using a sphere formation assay protocol previously designed by our group [41], we evaluated the stem/progenitor cell-like properties of both cell lines upon exposure to either of the two treatments. Single cell suspensions of PLum-AD and PLum-AI were embedded in Matrigel™ and maintained for 10 to 12 days. On the last day, prostatospheres were visualized and counted under an inverted light microscope and the sphere formating unit (SFU) as well as the average size of generated spheres in the different treatment conditions were calculated (Fig 5).

Our results showed that both the number and size of spheres decreased significantly in both cell lines upon treatment with SB203580 (Fig 5B and 5C). Sphere formation dropped significantly from 10.76 ± 0.57% to 8.01 ± 1.85% (p-value < 0.05) in PLum-AD and from 10.7 ± 0.09% to 8.49 ± 0.17% (p-value < 0.001) in PLum-AI SB203580-treated cells (Fig 5B). Moreover, SB203580 significantly reduced the diameter of prostatospheres formed in PLum-AD (98.64 ± 2.83 μm to 80.85 ± 2.74 μm in the treated group; p-value < 0.001) and PLum-AI (114.85 ± 3.18 μm to 95.5 ± 2.5 μm in the treated group; p-value < 0.001) cells (Fig 5A and 5C).

On the other hand, upon treatment with IL-6, a significant increase was observed in the SFUs of both cell lines (12.55 ± 0.37 and 11.57 ± 0.16 in PLum-AD and PLum-AI; p-value < 0.05 and < 0.001 respectively) (Fig 5B). Similarly, IL-6 significantly and prominently increased the average diameter of PLum-AD spheres to 123.05 ± 4.35% (p-value < 0.001) and to 124.05 ± 2.96% in PLum-AI spheres (p-value < 0.05) (Fig 5A and 5C). Our results show that SB203580 decreases the stem/progenitor cell subpopulation in PLum-AD and PLum-AI cells evident by a decrease in the number and size of formed spheres compared to control; whereas, IL-6 promoted the growth and survival of this subpopulation.

## Discussion

Progression of PCa from primary stages to advanced and metastatic CRPC signifies an important sector of the management of this debilitating disease due to its high mortality rates and increasing incidence around the world [1, 49, 50]. Despite the efforts that were made during the past few years to identify new molecular targets and develop targeted agents for CRPC, majority of those therapies have failed clinically. This is due to our poor understanding of the subcellular mechanisms underlying the process of PCa progression, making detection difficult for the early stages of this disease. In an attempt to fill this void, our group interrogated molecular “oncophenotypes” that embody the transition of PCa from an androgen-dependent (AD) to–independent (AI) states [8]. In this study, we worked on identifying phenotypic and expression cues that underly the augmented aggressiveness and malignant phenotype of PCa and shedding light on new mechanisms pertaining to this progression as well as potential therapeutic vulnerabilities for CRPC, using our previously established murine cell models of PCa [8].

Our group has previously generated an *in vitro* murine PCa cell model, namely Prostate Luminal (PLum) cells, that harbor a *Pten*^-/-^*TP53*^-/-^ genetic background [38]. Later, our group modified these cells to represent the progression of PCa from a primary to an advanced stage by conditioning PLum cells to survive under androgen-rich or -deprived milieus. Thus, cells that were grown under androgen-dependent conditions and which represent the primary stage of PCa are now termed PLum-AD cells, whereas cells that were grown under androgen-independent conditions and which represent advanced mCRPC are termed PLum-AI cells. In the absence of reliable *in vitro* cell culture models of PCa recapping primary adenocarcinoma and progressive androgen-independent disease, our models embody potentials to aid in better understanding the molecular cues involved in PCa progression via gene profiling analysis. More importantly, since our two murine cell models were isolated from *Pten*^-/-^*TP53*^-/-^ tumors, the intra-tumor heterogeneity (ITH) that a prostate tissue normally holds, and which should be accounted for in each context or model is then eliminated [38]. Therefore, our models providentially carry a significant indication of the purity of cell lines as they represent a homogenous population of cells, with PLum-AD representing primary PCa cells and displaying an epithelial phenotype and PLum-AI representing hormone-refractory PCa and harboring a mesenchymal cell morphology.

We used a previously generated microarray data from our group [8] and performed GSEA to evaluate differential gene expression at the level of gene sets. GSEA posits a powerful analytical tool for interpreting gene expression data and extracting meaningful insights into pathobiological mechanisms and molecular pathways in the context of diseases [51]. Our data revealed the enrichment of biological functions and pathways in PLum-AI cells that are central to tumor aggressiveness, which mainly contribute to EMT and cell migration and invasion. Interestingly, our results identified a list of genes that are differentially expressed in PLum-AI compared to PLum-AD cells, among which were *Cdh1*, *Tnf*, *Egf*, *NF-κB*, *p38* and *Il-6*.

Our results demonstrated that *p38* was predicted to be significantly upregulated in PLum-AI vs. PLum-AD cells. Conversely, gene set analysis showed that gene sets which have been previously associated with favorable response to the p38 inhibitor, SB203580, were attenuated (i.e., inversely enriched) in PLum-AI cells, thus suggesting that these aggressive cells may be therapeutically vulnerable to p38 inhibition. Gene network analysis showed that the p38 MAPK inhibitor SB203580 constrains numerous genes that contribute to signaling networks associated with inflammation and immune function/response including, but not limited to *Tnf*, *Il-6*, *Mmp13*, *Twist*, and *Ccl2*. Therefore, we decided to utilize this treatment and evaluate its effect on both cell lines. According to GSEA, inhibiting p38 in PLum-AI cells will induce MErT, thereby upregulating epithelial markers and downregulating mesenchymal markers. A review of the literature unveiled significant evidence of the invasive and metastatic effects of Ras oncogenes and Ras signaling pathways in PCa [52, 53]. Accordingly, findings of the involvement of p38 in the acquisition of metastatic properties in PCa further supported our predictions [54].

According to the gene network analysis, SB203580 inhibits several downstream genes involved in the aggressive characteristics of PLum-AI cells and therefore in PCa progression. Some of these genes were *Ptges*, *Ctgf*, *Vim*, *Mmp2*, *Ccl2*, *Ar* and its direct target *p38*. SB203580 was also revealed to upregulate the expression of epithelial markers such as *Cdh1* and *Cd40*. These results were validated on PLum-AI cells using qRT-PCR. The selected genes were chosen based on the shared signaling pathways between SB203580 and IL-6, the two treatments of interest, as well as their pivotal biological purpose that grants PLum-AI their aggressive and metastatic behavior. Our enrichment and qRT-PCR results rhyme with findings in literature. Interestingly, the two cytoplasmic markers, *Vim* and *Cdh1*, showed the most sensitivity to SB203580. The p38 MAPK inhibitor significantly repressed the mesenchymal marker *Vim* and augmented the epithelial marker *Cdh1*, encoding for E-Cadherin. Correlation between PCa and epithelial/mesenchymal markers has been discovered and investigated for several decades. Vimentin is a mesenchymal marker often recorded in tumor cell invasions and correlated with EMT [45, 55]. Whereas the expression of E-cadherin behaved to proctor the epithelial morphology; its loss is considered an indicator of EMT [55]. When it comes to the relationship between p38 and these surface markers, few reports emerged in the past few years revealing that reduced phosphorylation of p38 leads to the downregulation of several EMT-markers such as VIM and CDH2 in LNCaP cells [56]. Another recent report focused on the suppression of E-cadherin and induction of the EMT program through activating the p38-FOXC2-ZEB1 axis, knowing that this axis has a direct repressing effect on CDH1 in breast cancer cells [57]. The transcriptional suppression by ZEB proteins on E-cadherin was evidenced in PCa by Graham *et al*., but the p38-FOXC2-ZEB1 axis has not been previously studied in PCa [58, 59]. Therefore, to the best of our knowledge, we are hereby reporting, for the first time, the effect of p38 suppression on *Cdh1* in PCa.

We selected growth factors and enzymes that are affected by the inhibition of p38, based on common genes between SB203580 and IL-6 mechanistic networks, in order to validate by qRT-PCR. Particularly, we validated the overexpression of three genes, that are deemed as key members in metastasis and progression of PCa, upon IL-6 treatment, namely *Mmp2*, *Ptges* and *Ctgf*. The same genes were shown to be under-expressed after SB203580 treatment. Matrix metalloproteinase 2 (Mmp2) is an important member of the matrix metalloproteinases (Mmp) family that affects collagen junctions between cells. Overexpression of Mmp2 prompts the disassembly of the basement membrane and facilitates metastasis [46]. Our results confirmed that *Mmp2* is a downstream regulator of p38 MAPK and its overexpression plays an important role in tumor invasion. Prostaglandin E synthase (*Ptges*) enriches for numerous mechanisms that endorse cancer growth and progression, such as amplified proliferation, migration and invasion, angiogenesis, counteracted apoptosis, and inflammation [44]. PTGES expression has been described to be upregulated by p38 in osteoarthritic human cartilage [60] and in microglia [61], but no evidence in prostate cells has been reported. The upregulation of connective tissue growth factor (*Ctgf*) in PCa cells was reported to promote angiogenesis and tumor growth [47].

CD40 is a prominent member of the TNF receptor superfamily and is widely expressed on immunocytes and frequently on epithelial malignancies. Palmer *et al*. showed CD40 to be more expressed in normal prostatic acini or primary PCa versus more advanced PCa tissues, suggesting that invasive PCa is a CD40-negative tumor [43]. Our findings of an increase in *Cd40* expression in PLum-AI cells upon p38 inhibition further confirm past findings and suggest that *Cd40* expression is inversely correlated with PCa progression. Monocyte chemoattractant protein 1 (CCL2) is a member of the CC chemokine family and is known to promote monocyte recruitment to sites of inflammation. It has been implied in the activation of the PI3 kinase/Akt signaling pathway and subsequent PCa progression and metastasis to the bone microenvironment [62, 63]. Here, we demonstrated reduced expression of *Ccl2* with SB203580 treatment and its increase with IL-6 in PLum-AI and PLum-AD cells respectively.

Studies have elucidated the importance of AR in the development and progression of PCa, which is the basis for androgen deprivation therapy (ADT), the first line of treatment of localized PCa [64]. However, ultimately PCa changes AR and adapts to survive under castration levels of androgen through different mechanisms that overexpression and amplification of the androgen receptor or through mutations of the receptor to a constitutively active form [3]. Therefore, the aforementioned qRT-PCR validation data suggests that our murine PLum-AI cells, in contrast to PLum-AD cells, exhibit molecular features of CRPC.

In this study, we also aimed to identify other molecular cues that could help us better understand the subcellular pathways underlying PCa progression from a primary localized to a metastatic stage. Based on the gene-network analysis, we identified IL-6 to be heavily inhibited by the p38 inhibitor SB203580. This is complimentary to a previous study addressing reduced IL-6 production by p38 inhibition in normal prostate [65]. Herein, we applied recombinant human IL-6 protein on PLum-AD and validated its effect using qRT-PCR. While SB203580 inhibited *Vim*, *Mmp2*, *Ptges*, and *Ctgf*, and activated *Cdh1*, IL-6 posed an opposing effect thus upregulating the expression of these genes and activating important inflammatory signaling pathways. This is in concordance with evidence from literature that points to massive inflammatory input in the tumor microenvironment [11].

To further assess the phenotypic changes accompanied by the alteration of expression of the mentioned genes, we investigated the effect of SB203580 and IL-6 on the morphology of the cells. Our results revealed that SB203580 induced MErT in PLum-AI thus converting them into epithelial colonies by the upregulation of CK8 and downregulation of Vim. Our results are in concordance with previous reports that suggested an upregulation of E-Cadherin and induction of MErT upon p38 suppression [39, 66]. Also, we found that p38 inhibition is involved in hindering PCa cell migration, as evident by the efficient inhibition of migration of PLum-AD and PLum-AI cells with the p38 inhibitor SB203580 using the wound healing and the trans-well migration assays. A recent paper investigating the contribution of CCL21/CCR7 signaling in lymph node metastasis reported the same results and deduced that p38 upregulation is involved in CCL21‐induced PCa cell migration [67]. Another report recorded the same inhibitory effect of SB203580 on cell migration of breast cancer cell lines [68] and human PCa cell lines [66].

Also, the tumorigenic effect of IL-6 on the morphology, proliferation and migration of cells was validated phenotypically. We demonstrated the enhanced pro-proliferative and migratory behavior of PLum-AD and PLum-AI upon exposure to IL-6, supplementing our earlier findings on the role of IL-6 in promoting EMT and enhancing cell survival. These results overlap with the findings of a recent report concluding the effect of IL-6 in promoting resistance to ADT and therefore the transformation of primary PCa to mCRPC [69].

Knowing that sphere formation assay can be utilized as a functional indicator of the progenitor activity, differentiation, and self-renewal ability of the stem/progenitor cell population [41, 70], we utilized this method to investigate the ability of SB203580 and IL-6 to target this sub-population of cells in PLum-AD and PLum-AI. The importance of this 3D formation cell culture system is that it permits cell growth in a more physiologically relevant environment, giving it advantage over 2D cell cultures [71, 72]. Treatment with SB203580 at a concentration of 10 μM significantly inhibited sphere formation as well as the size of spheres in both cell lines. Thus, SB203580 might serve as a potential therapy in targeting the self-renewal ability of PCa CSCs, eliminating the whole tumor and preventing recurrence [66]. Suggestions of surpassing therapy resistance in aggressive PCa by co-treating with SB203580 and other chemotherapies such as Docetaxel and Enzalutamide had shown a promising desensitization of cancer cells to the drug [66]. When it comes to the favorable effect of IL-6 on the stem/progenitor cell population, our results suggested an increase in the number and size of PLum-AD and PLum-AI spheres. A study in 2011 revealed that IL-6 is essential in converting non-stem cancer cells to CSCs in human prostate and breast cell lines, and that CSCs express and release naturally higher levels of IL-6 than their non-stem counterparts, thus promoting tumor aggressiveness [73].

### Study limitations

We believe our study has several limitations. First, we did not analyze in our study the putative interactions between p38/MAPK, IL-6, PTEN and/or p53 signaling, and therefore any links between these pathways in our model remain to be established. In fact, as future directions, we plan to apply anti-IL-6 on PLum-AI cells and assess its effect as a sole treatment on PCa progression, or in combination with SB203580 and other traditional and in-clinical-trial PCa drugs, and further validate these findings using syngeneic *in vivo* mouse models. *In vivo* studies are of importance since IL-6 protein is a cytokine that is mostly released by myeloid cells. Second, since IL-6 treatment and p38 inhibition might impinge on separate pathways, combinatorial treatment would be of interest to further decipher the interplay between both molecular mechanistic pathways and assess how p38 inhibition affects IL-6 and how IL-6 treatment impacts p38 phosphorylation and other MAPKs. Indeed, knocking down or over-expressing one of these targets downstream of treating with IL-6 or p38 inhibition would further highlight the molecules that may connect both IL-6 and p38 pathways and their relevance to PCa progression. Third, data presented in our study showed three cell populations displaying an epithelial (CK8), mesenchymal (Vim), and a mixed phenotype (CK8/Vim). Henceforth, it is interesting to sort out the different cell populations and characterize them independently. Fourth, regulation of E-cadherin, androgen receptor, p38 activity, c-Jun N-terminal Kinase (JNK) activation, and other stem cell and neuroendocrine markers by SB203580 and IL-6 could be further confirmed using syngeneic *in vivo* mouse models and at the protein level *in vitro* in future studies.

## Conclusions

**Fig 6:**
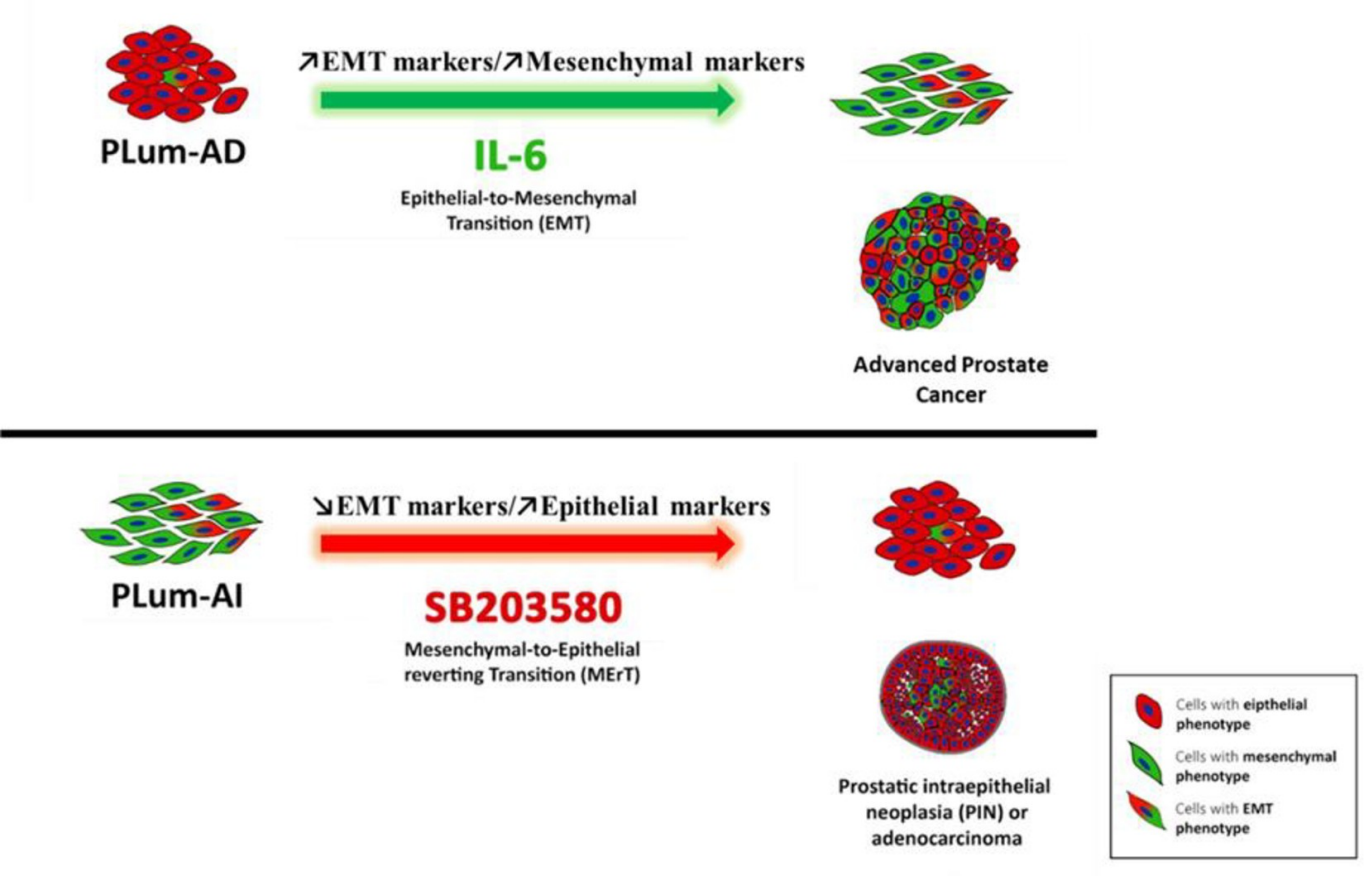
Schematic model showing phenotypic progression of PCa from primary adenocarcinoma (presented by PLum-AD cells) to advanced CRPC (presented by PLum-AI cells) and the effects of SB203580 and IL-6 on this progression. PLum-AD cells which demonstrate an epithelial phenotype undergo EMT following the exposure to IL-6, whereas Plum-AI cells which exhibit a mesenchymal phenotype undergo MErT upon treatment with the p38 MAPK inhibitor SB203580.

To conclude, we identified differentially expressed genes in PLum-AI compared to PLum-AD cells. We enriched for pathways underlying this transition using GSEA method, then identified high-potential therapeutic targets. As a matter of proof, we validated the gene-profiling results on the molecular and functional levels. We also confirmed the therapeutic vulnerability of PLum-AD and PLum-AI to p38 inhibition, and the aggressive feature that both cell lines acquire with IL-6 (Fig 6). Our findings shed light on the remarkable representation of PCa progression that these two cell lines recapitulate, and the advantages they carry over cell lines of different backgrounds. Additionally, p38 inhibition proved promising results needing further investigation, especially upon subsequent co-treatment with other available therapies.

## Supporting information

S1 Table Different pairs of primers used in RT-qPCR. (DOCX)

S2 Table Representative upstream regulators (with a negative z-score) of PLum-AI vs. PLum-AD resulting from the Gene Set Enrichment Analysis (GSEA) of transcriptomes, and their role in PCa. GSEA was performed on n = 3 samples from each cell line revealing 723 differentially expressed genes in PLum-AI vs. PLum-AD cells. PLum-AI cells showed inhibition of the p38 inhibitor SB203580.
(DOCX)

S3 Table Representative upstream regulators (with a positive z-score) of PLum-AI vs. PLum-AD resulting from the Gene Set Enrichment Analysis (GSEA) of transcriptomes, and their role in PCa. GSEA was performed on n = 3 samples from each cell line revealing 723 differentially expressed genes in PLum-AI vs. PLum-AD cells. PLum-AI cells showed activation of signaling networks associated with EMT, inflammation and immune response, such as EGF, IL-6, LPS, and SP1.
(DOCX)
