# Supplementary material for: Genome-wide gene expression analysis of a murine model of prostate cancer progression: Deciphering the roles of IL-6 and p38 MAPK as potential therapeutic targets
Source: PLoS One. 2020 Aug 13;15(8):e0237442. doi: 10.1371/journal.pone.0237442 (PMC7425932; doi:10.1371/journal.pone.0237442)
Supplement: S1 Table — (DOCX) [file pone.0237442.s001.docx]

**S1 Table.** Different pairs of primers used in RT-qPCR

| **Murine gene** | **Primer Sequence (5’ – 3’)** |
| --- | --- |
| *Gapdh* | F- CAGAACATCATCCCTGCATC |
|  | R- CTGCTTCACCACCTTCTTGA |
| *Cdh1* | F- AGGTCTCCTCATGGCTTTGC |
|  | R- GTCTCCAGCTTGTGGAGCTT |
| *Ctgf* | F- GCGCCTGTTCTAAGACCTGT |
|  | R- AGGTGTCCGGATGCACTTTT |
| *Vim* | F- TGAGATCGCCACCTACAGGA |
|  | R- GAGTGGGTGTCAACCAGAGG |
| *Ptges* | F- GCTGTCATCACAGGCCAGAT |
|  | R- GGTTGGGTCCCAGGAATGAG |
| *Mmp2* | F- ACAACAGCTGTACCACCGAG |
|  | R- GAAGGGGAAGACACATGGGG |
| *p38* | F- CGCAAGGTCACTGGAGGAAT |
|  | R- CTGGGCTTTAGGTCCCTGTG |
| *Ar* | F- GACTCTGGGAGCTCGTAAGC |
|  | R- ACTCCTGGCTCAATGGCTTC |
| *Ccl2* | F- ACAAGAGGATCACCAGCAGC |
|  | R- GGACCCATTCCTTCTTGGGG |
| *Cd40* | F- ATGCCACCCATGTGACTCAG |
|  | R- GGTGCCCTCCTTCTTAACCC |
