# Supplementary material for: Genome-wide gene expression analysis of a murine model of prostate cancer progression: Deciphering the roles of IL-6 and p38 MAPK as potential therapeutic targets
Source: PLoS One. 2020 Aug 13;15(8):e0237442. doi: 10.1371/journal.pone.0237442 (PMC7425932; doi:10.1371/journal.pone.0237442)
Supplement: S2 Table — GSEA was performed on n = 3 samples from each cell line revealing 723 differentially expressed genes in PLum-AI vs. PLum-AD cells. PLum-AI cells showed inhibition of the p38 inhibitor SB203580. (DOCX) [file pone.0237442.s002.docx]

**S2 Table.** **Representative upstream regulators (with a negative z-score) of PLum-AI vs. PLum-AD resulting from the Gene Set Enrichment Analysis (GSEA) of transcriptomes, and their role in PCa.** GSEA was performed on n=3 samples from each cell line revealing 723 differentially expressed genes in PLum-AI vs. PLum-AD cells. PLum-AI cells showed inhibition of the p38 inhibitor SB203580.

| **Upstream Regulator** | **Activation z-score** | **Role in PCa** |
| --- | --- | --- |
| **Alpha catenin** | -3.871 | Down-regulation has been correlated to several malignant cellular features [1] |
| **SB203580** | -3.461 | SB203580 is a p38 MAPK inhibitor. It is overexpressed in well- and moderately- differentiated PCa [2] |
| **Sirolimus** | -2.843 | Sirolimus (Rapamycin) is an mTORC1 inhibitor, which inhibits the PI3K/Akt/mTOR signaling pathway. It has been proven that it is commonly altered in PCa [3]. |
| **Pyrrolidine dithiocarbamate** | -2.829 | Pyrrolidine dithiocarbamate is an inhibitor of NF-κB signaling, which plays a critical role in cancer cell invasion and metastasis [4]. |
| **PRKAA2** | -2.728 | It has been shown that loss of PRKAA2 causes the tumours to grow more rapidly. No evidence on its role in PCa specifically [5]. |
| **Flutamide** | -1.976 | Flutamide is an AR antagonist, the latter which plays a critical role in PCa by transactivation of multiple genes involved in tumorigenesis [6]. |
| **NR1H2** | -1.974 | NR1H2 plays a pivotal role in both prostate homeostasis and carcinogenesis [7]. |
| **FTY720** | -1.972 | FTY720 modulates estrogenic micromilieu and interrupts its cross talk with sphingolipid metabolism [8]. |
| **Apigenin** | -1.969 | Apigenin is a natural flavone, that has been used as a chemopreventive agent and treatment for PCa via inhibition of androgen production [9]. |
| **SLPI** | -1.969 | SLPI is regulated by AR in androgen-independent behavior in CRPC cells. It is required for CRPC cell proliferation under androgen-deprived conditions [10]. |

**Abbreviations:** PRKAA2: AMP-activated protein kinase (AMPK) gene encoding for the α2 isoform; NR1H2: nuclear receptor subfamily 1 group H member 2; FTY720: fingolimod; SLPI: secretory leukocyte peptidase inhibitor; AR: androgen receptor; CRPC: castration-resistant prostate cancer; PCa: prostate cancer.

**S2 Table References:**

1. Aaltomaa S, Lipponen P, Ala-Opas M, Eskelinen M, Kosma VM. Alpha-catenin expression has prognostic value in local and locally advanced prostate cancer. British journal of cancer. 1999;80(3-4):477-82. Epub 1999/07/17. doi: 10.1038/sj.bjc.6690381. PubMed PMID: 10408856; PubMed Central PMCID: PMCPMC2362305.

2. Uzgare AR, Kaplan PJ, Greenberg NM. Differential expression and/or activation of P38MAPK, erk1/2, and jnk during the initiation and progression of prostate cancer. The Prostate. 2003;55(2):128-39.

3. Imrali A, Mao X, Yeste-Velasco M, Shamash J, Lu Y. Rapamycin inhibits prostate cancer cell growth through cyclin D1 and enhances the cytotoxic efficacy of cisplatin. American journal of cancer research. 2016;6(8):1772-84. Epub 2016/09/21. PubMed PMID: 27648364; PubMed Central PMCID: PMCPMC5004078.

4. Yang JR, Pan TJ, Yang H, Wang T, Liu W, Liu B, et al. Kindlin-2 promotes invasiveness of prostate cancer cells via NF-kappaB-dependent upregulation of matrix metalloproteinases. Gene. 2016;576(1 Pt 3):571-6. Epub 2015/11/10. doi: 10.1016/j.gene.2015.11.005. PubMed PMID: 26551397.

5. Ross FA, MacKintosh C, Hardie DG. AMP-activated protein kinase: a cellular energy sensor that comes in 12 flavours. The FEBS journal. 2016;283(16):2987-3001. Epub 2016/03/05. doi: 10.1111/febs.13698. PubMed PMID: 26934201; PubMed Central PMCID: PMCPMC4995730.

6. Cui K, Li X, Du Y, Tang X, Arai S, Geng Y, et al. Chemoprevention of prostate cancer in men with high-grade prostatic intraepithelial neoplasia (HGPIN): a systematic review and adjusted indirect treatment comparison. Oncotarget. 2017;8(22):36674-84. Epub 2017/04/19. doi: 10.18632/oncotarget.16230. PubMed PMID: 28415774; PubMed Central PMCID: PMCPMC5482687.

7. Bousset L, Rambur A, Fouache A, Bunay J, Morel L, Lobaccaro JA, et al. New Insights in Prostate Cancer Development and Tumor Therapy: Modulation of Nuclear Receptors and the Specific Role of Liver X Receptors. International journal of molecular sciences. 2018;19(9). Epub 2018/08/30. doi: 10.3390/ijms19092545. PubMed PMID: 30154328; PubMed Central PMCID: PMCPMC6164771.

8. Allam RM, Al-Abd AM, Khedr A, Sharaf OA, Nofal SM, Khalifa AE, et al. Fingolimod interrupts the cross talk between estrogen metabolism and sphingolipid metabolism within prostate cancer cells. Toxicology letters. 2018;291:77-85. Epub 2018/04/15. doi: 10.1016/j.toxlet.2018.04.008. PubMed PMID: 29654831.

9. Wang X, Wang G, Li X, Liu J, Hong T, Zhu Q, et al. Suppression of rat and human androgen biosynthetic enzymes by apigenin: Possible use for the treatment of prostate cancer. Fitoterapia. 2016;111:66-72. Epub 2016/04/23. doi: 10.1016/j.fitote.2016.04.014. PubMed PMID: 27102611.

10. Zheng D, Gui B, Gray KP, Tinay I, Rafiei S, Huang Q, et al. Secretory leukocyte protease inhibitor is a survival and proliferation factor for castration-resistant prostate cancer. Oncogene. 2016;35(36):4807-15. Epub 2016/02/16. doi: 10.1038/onc.2016.13. PubMed PMID: 26876202.
