# Supplementary material for: Genome-wide gene expression analysis of a murine model of prostate cancer progression: Deciphering the roles of IL-6 and p38 MAPK as potential therapeutic targets
Source: PLoS One. 2020 Aug 13;15(8):e0237442. doi: 10.1371/journal.pone.0237442 (PMC7425932; doi:10.1371/journal.pone.0237442)
Supplement: S3 Table — GSEA was performed on n = 3 samples from each cell line revealing 723 differentially expressed genes in PLum-AI vs. PLum-AD cells. PLum-AI cells showed activation of signaling networks associated with EMT, inflammation and immune response, such as EGF, IL-6, LPS, and SP1. (DOCX) [file pone.0237442.s003.docx]

**S3 Table. Representative upstream regulators (with a positive z-score) of PLum-AI vs. PLum-AD resulting from the Gene Set Enrichment Analysis (GSEA) of transcriptomes, and their role in PCa.** GSEA was performed on n=3 samples from each cell line revealing 723 differentially expressed genes in PLum-AI vs. PLum-AD cells. PLum-AI cells showed activation of signaling networks associated with EMT, inflammation and immune response, such as EGF, IL-6, LPS, and SP1.

| **Upstream Regulator** | **Activation z-score** | **Role in PCa** |
| --- | --- | --- |
| **LPS** | 4.329 | LPS is considered a strong inflammatory stimulator and has been associated with chronic inflammation. Exposure to LPS can induce PCa migration and metastasis by encouraging the secretion of TNF-α and IL-6 [1]. |
| **IL-6** | 3.791 | IL-6 promotes cell survival and growth, besides indorsing the development of CRPC through several mechanisms as previously discussed. |
| **IKBKB** | 3.512 | IKBKB has a key role in both EMT and apoptosis of PCa [2]. |
| **SP1** | 3.437 | SP1 is overexpressed in CRPC and modulates metabolism and autophagy in aggressive PCa [3]. |
| **CEBPB** | 3.222 | CEBPB expression has been shown to be increased in tumor samples compared to normal samples, which infers their association with gain of regulatory control in cancer (possibly turning on of oncogenes or other tumorigenic mechanisms) [4]. |
| **forskolin** | 2.500 | Treating LNCaP PCa cells with Forskolin upregulates AR expression and activity (via PSA induction) [5]. |
| **EGF** | 2.495 | EGF evokes EMT and PCa cell progression by inducing HIF-1α expression and morphological changes from epithelial to mesenchymal phenotypes [6]. |
| **MAPK1** | 2.469 | MAPKs play pivotal roles in regulating cancer cell invasion and metastasis [7]. |
| **LEP** | 2.460 | LEP contributes to cancer growth and development via stimulating inflammation, angiogenesis, proliferation and EMT [8]. |
| **MEK** | 2.452 | MEK is incorporated in several signaling cascades dysregulated in PCa, and hence it is upregulated herein [9]. |

**Abbreviations:** LPS: lipopolysaccharide; PCa: prostate cancer; TNF: tumor necrotic factor; CRPC: castration-resistant prostate cancer; IKBKB: Inhibitor of Nuclear Factor Kappa B Kinase Subunit Beta; SP1: transcriptional factor specificity protein 1; CEBPB: CCAAT/Enhancer binding protein β; AR: androgen receptor; PSA: prostate specific antigen; EGF: epidermal growth factor; EMT: epithelial-mesenchymal transition; LEP: Leptin; MAPK: mitogen-activated protein kinase; MEK: MAPK kinase

**S3 Table References:**

1. Xu P, Cai F, Liu X, Guo L. Sesamin inhibits lipopolysaccharide-induced proliferation and invasion through the p38-MAPK and NF-kappaB signaling pathways in prostate cancer cells. Oncology reports. 2015;33(6):3117-23. Epub 2015/04/08. doi: 10.3892/or.2015.3888. PubMed PMID: 25845399.

2. Ping H, Yang F, Wang M, Niu Y, Xing N. IKK inhibitor suppresses epithelial-mesenchymal transition and induces cell death in prostate cancer. Oncology reports. 2016;36(3):1658-64. Epub 2016/07/20. doi: 10.3892/or.2016.4915. PubMed PMID: 27432067.

3. Ling Z, Liu D, Zhang G, Liang Q, Xiang P, Xu Y, et al. miR-361-5p modulates metabolism and autophagy via the Sp1-mediated regulation of PKM2 in prostate cancer. Oncology reports. 2017;38(3):1621-8. Epub 2017/11/03. doi: 10.3892/or.2017.5852. PubMed PMID: 29094170.

4. Reddy A, Huang CC, Liu H, Delisi C, Nevalainen MT, Szalma S, et al. Robust gene network analysis reveals alteration of the STAT5a network as a hallmark of prostate cancer. Genome informatics International Conference on Genome Informatics. 2010;24:139-53. Epub 2010/07/01. PubMed PMID: 22081596; PubMed Central PMCID: PMCPMC6035043.

5. Desiniotis A, Schäfer G, Klocker H, Eder IE. Enhanced antiproliferative and proapoptotic effects on prostate cancer cells by simultaneously inhibiting androgen receptor and cAMP-dependent protein kinase A. International Journal of Cancer. 2010;126(3):775-89. doi: doi:10.1002/ijc.24806.

6. Cho KH, Choi MJ, Jeong KJ, Kim JJ, Hwang MH, Shin SC, et al. A ROS/STAT3/HIF-1alpha signaling cascade mediates EGF-induced TWIST1 expression and prostate cancer cell invasion. Prostate. 2014;74(5):528-36. Epub 2014/01/18. doi: 10.1002/pros.22776. PubMed PMID: 24435707.

7. Li Y, Luo H, Xiao N, Duan J, Wang Z, Wang S. Long Noncoding RNA SChLAP1 Accelerates the Proliferation and Metastasis of Prostate Cancer via Targeting miR-198 and Promoting the MAPK1 Pathway. Oncology research. 2018;26(1):131-43. Epub 2017/05/12. doi: 10.3727/096504017x14944585873631. PubMed PMID: 28492138.

8. Hu MB, Xu H, Hu JM, Zhu WH, Yang T, Jiang HW, et al. Genetic polymorphisms in leptin, adiponectin and their receptors affect risk and aggressiveness of prostate cancer: evidence from a meta-analysis and pooled-review. Oncotarget. 2016;7(49):81049-61. Epub 2016/10/22. doi: 10.18632/oncotarget.12747. PubMed PMID: 27768592; PubMed Central PMCID: PMCPMC5348375.

9. Park H, Kim Y, Sul JW, Jeong IG, Yi HJ, Ahn JB, et al. Synergistic anticancer efficacy of MEK inhibition and dual PI3K/mTOR inhibition in castration-resistant prostate cancer. Prostate. 2015;75(15):1747-59. Epub 2015/08/08. doi: 10.1002/pros.23057. PubMed PMID: 26250606.
